# MiR-382 functions as tumor suppressor and chemosensitizer in colorectal cancer

**DOI:** 10.1042/BSR20180441

**Published:** 2019-08-09

**Authors:** Hui Yao, Dong Xia, Zong-lin Li, Lei Ren, Ming-ming Wang, Wang-sheng Chen, Zheng-chuan Hu, Guo-ping Yi, Liang Xu

**Affiliations:** Department of Gastrointestinal Surgery, The Affiliated Hospital of Southwest Medical University, Luzhou City, Sichuan Province 646000, China

**Keywords:** colorectal cancer, CDDP resistance, HIPK3, KLF12 signaling, miR-382

## Abstract

Increasing evidence suggests that microRNAs (miRNAs) play a critical role in tumorigenesis. Decreased expression of miR-382 has been observed in various types of cancers. However, the biological function of miR-382 in colorectal cancer (CRC) is still largely unknown. Here, we found that miR-382 was down-regulated in human colorectal cancer tissues and cell lines associated with it. MiR-382 inhibited colorectal cancer cell proliferation, migration, invasion, and enhance chemosensitivity. Furthermore, we identified Krüppel-like factor 12 (KLF12) and homeodomain-interacting protein kinase 3 (HIPK3) as the target of miR-382, and miR-382 rescued the promotion effect of KFL12 on migration and enhanced chemosensitivity in colorectal cancer cell lines. Collectively, these findings revealed that miR-382 inhibits migration and enhances chemosensitivity by targeting KLF12 and HIPK3 in colorectal cancer. These findings might serve as a tumor suppressor in CRC.

## Introduction

Colorectal cancer (CRC) is the third most prevalent malignant cancer worldwide, accounting for 1.2 million new diagnoses each year, and the third leading cause of cancer related death worldwide, accounting for 600,000 deaths every year [1]. Many risk factors contribute to the incidence of CRC, such as obesity, age, alcohol abuse, and genetic mutations [2]. The survival rate of CRC patients is still unsatisfied, even though the diagnostic and therapeutic option have been significantly improved [3]. Once metastasis has occurred, chemotherapy becomes the mainstay of treatment, but chemoresistance eventually limits the treatment effectiveness, then new targets are needed. Unfortunately, the mechanisms inhibit tumor growth and enhance chemosensitivity are not completely understood.

MicroRNAs (miRNAs) are 20–22 nucleotides in length and classified as small, noncoding RNAs. These small noncoding RNAs are known to regulate protein expression at the post-transcriptional level by binding the 3′-UTR portion of mRNAs to either prevent translation or promote the degradation of the mRNA [4], and it plays a crucial role in various physiological and pathological processes such as cellular differentiation, proliferation, apoptosis, and angiogenesis [5,6]. Once miRNA has been reported to be involved in the pathological processes of proliferation, metastasis, and drug resistance in multiple types of cancer, including breast, osteosarcoma, colorectal, and ovarian cancers [7–10], it has been named microRNA-382. We found that miR-382 was significantly down-regulated in CRC patients with cancer metastasis. Therefore, in conjunction with relevant literature [10], our results indicate that low levels of miR-382 may contribute to CDDP resistance and development of CRC. However, the possible roles and mechanisms of miR-382 in human CRC are still not well established.

In the present study, the expression levels and potential roles of miR-382 on human CRC were evaluated. The results illustrated that the expression levels of miR-382 were remarkably down-regulated in human CRC tissue specimens and cell lines when compared with their adjacent normal tissues and control cell line. Furthermore, enforced expression of miR-382 significantly suppressed CRC cell proliferation and enhanced chemosensitivity by directly targeting Krüppel-like factor (KLF) 12 and HIPK3. These findings might provide reliable evidence for the development of novel therapeutics for human CRC treatment.

## Materials and methods

### Tissue sample collection

CDDP-sensitive and -tolerant colorectal cancer samples were obtained from the Affiliated Hospital of Southwest Medical University. Written informed consent was obtained from the patients, and the study was approved by Institutional Human Experiment and Ethics Committee of the Affiliated Hospital of Southwest Medical University.

### Cell culture

LS1034 and HCT116 colorectal cancer cells were maintained in RPMI1640, Eagle’s Minimum Essential Medium and Dulbecco’s modified Eagle’s medium supplemented with 10% fetal bovine serum and 1% antibiotic–antimycotic solution (100 U/ml penicillin and 100 μg/ml streptomycin) respectively. Cells were in a water-jacketed CO_2_ incubator at 37°C and 5% CO_2_. Transfection was performed with Lipofectamine™ 2000 (Invitrogen, Carlsbad, CA, U.S.A.) as per the manufacturer’s instructions.

### RNA extraction and quantitative real-time PCR

Total RNA was extracted using TRIzol reagent (Life Technologies) according to the recommended protocols of the manufacturer. First-strand cDNA was synthesized by reverse transcription. Glyceraldehyde-3- phosphate dehydrogenase (GAPDH) or U6 small nuclear RNA was used as the internal reference.

For miR-382 expression, a stem–loop reverse transcription-polymerase chain reaction (RT-PCR) was carried out using an All-in-One™ miRNA quantitative RT-PCR (qRT-PCR) Detection Kit (Gene Copoeia, Rockville, MD, U.S.A.). The primer sequences for genes were defined as follows: KLF12 forward 5′-CACCTGGAAATG TGAACAACA-3′, reverse 5′-TTTTACTTTGTCTGGGAGATAGGC-3′; HIPK3 forward 5′-ACATTGGAAGAGCATGAGGCAGAGA-3′, reverse 5′-CTGCTG AAAAGCATCACCACAACCA-3′; GAPDH forward 5′-GCAGGGGGGAGCCAAAA GGGT-3′ and reverse 5′-TGGGTGGCAGTGATGGCATGG-3′. Real-time PCR was performed using a Bio-Rad iQ5 system, and the fold change in expression of each target RNA relative to U6 snRNA or GAPDH mRNA was calculated based on the threshold cycle (*C*_t_) as 2−(*C*_t_), where Δ*C*_t_ = *C*_t,target_ − *C*_t,U6_/_GAPDH_ and Δ (Δ*C*_t_) = Δ*C*_t,sample −_ Δ*C*_t,control_.

### Western blot analysis

Cells were lysed with RIPA buffer. The protein concentrations were determined using BCA Protein Assay kit (Beijingdingguo, China). Equal amounts of proteins from different samples were subjected to sodium dodecyl sulfate polyacrylamide gel electrophoresis, and the separated proteins were then transferred to a PVDF membrane. After blocking 1 h at room temperature, anti-KLF12, anti-HIPK3, and anti-β-actin antibodies were added and incubated at 4°C overnight. An HRP-conjugated secondary antibody was used, and the bands were visualized by chemiluminescence. Protein expression was quantified using Image-Lad 5.0 software (Bio-Rad, U.S.A.) normalized against internal control (β-actin).

### Cell proliferation assay

Cells well were plated into 96-well plates. After culturing for 24, 48, or 72 h, the cells were treated with 100 μl fresh serum-free medium containing 10 μl/well CCK-8 solution (Biosharp, China), according to the manufacturer’s protocol. Following incubation at 37˚C for 90 min, the absorbance was measured by microplate reader (Thermo, U.S.A.) at 570 nm at room temperature.

### Colony formation assay

Cells were seeded into 60 mm plates, then cells were cultured in growth medium containing 0.3% noble agar to allow formation of natural colonies. After 12 days, the plates were stained with Crystal Violet (Sigma) in 70% ethanol. The number of stained colonies was counted under a microscope (Olympus, Japan).

### Xenograft tumor formation assay

LS1034 cells were transfected with a corresponding plasmid. A total of 5 × 10^6^ cells in serum-free medium were injected s.c. into 6-week-old female athymic mice. The tumor weight was measured at the time that the mice were killed after 1 month injection.

Approximately 5 × 10^6^ LS1034 cells transfected with miR-382 mimics, inhibitor or negative control, with or without cisplatin treatment, were subcutaneously injected into the 6-week-old female athymic mice. The volume of Xenograft tumors was measured once per week until the mice were killed and during the course of the experiment, the mice were started on a treatment of either PBS or CDDP (10 mg/kg body weight). The tumor weight was measured at the time, when the mice were killed.

### Luciferase reporter assay

Wild-type HIPK3 and KLF12 3′-UTR containing a miR-382 binding site or mutant-form 3′-UTR was inserted downstream of the CDS of the firefly luciferase gene. The reporter gene was transfected into LS1034 or HCT116 cells with different miR-382 levels, simultaneously with Renilla luciferase. The activity of firefly luciferase or Renilla luciferase was measured 24 h post-transfection.

### Statistical analysis

All data are presented as the means ± SD. Differences between groups were determined using unpaired student’s *t*-test or one-way ANOVA using the SAS statistical software package version 6.12 (SAS Institute, Cary, NC). *P*-values less than 0.05 were considered statistically significant.

## Results

### Decreased expression of miR-382 contributes to CRC chemoresistance in *in vitro* and *in vivo*

**Figure 1:**
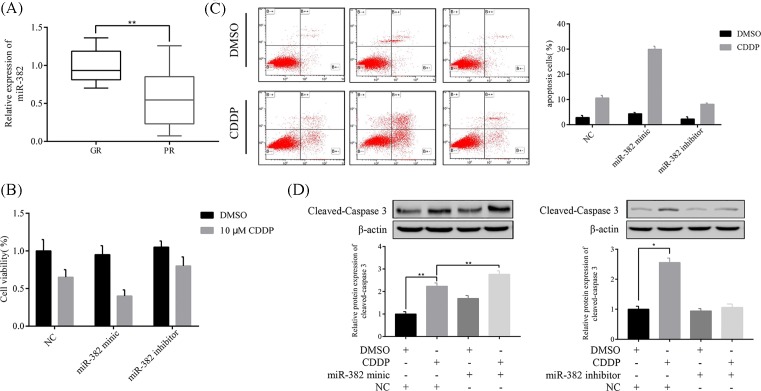
MiR-382 enhanced CDDP-induced apoptosis in CRC cell lines (**A**) MiR-382 expression significantly decreased in specimens of CRC patients that had a poor chemoresponse (PR, *n*=38) compared with those with a good chemoresponse (GR, *n*=64). (**B**) Overexpression of miR-382 increased CDDP-induced apoptosis in HCT116 cells. (**C**) Inhibition of miR-382 inhibited CDDP-induced apoptosis in HCT116 cells. After 24 h of transfection, cells were treated with DMSO or 10 μM CDDP for 24 h, followed by a flow cytometric assay. (**D**) Overexpression of miR-382 increased CDDP-induced caspase 3 cleavage, whereas inhibition of miR-382 suppressed CDDP-induced caspase 3 cleavage in HCT116 cells. All of the data are presented as the mean ± SD from three independent experiments; *P<0.05 and **P<0.01*.*

To investigate the correlation of miR-382 expression level and CDDP resistance in CRC patients, we measured the expression of miR-382 in 102 human CRC samples that acquired resistance to CDDP treatment compared with matched pretreatment samples (Figure 1A). Results show that in specimens of CRC patients that had a poor chemoresistance (PR = 38) compared with those that responded well to chemotherapy (GR = 64), miR-382 was significantly decreased. It means that miR-382 may be involved in CRC chemoresistance.

To identify whether miR-382 was related to CDDP resistance in CRC, we inhibit or overexpress miR-382 in CRC cells. As expected, CDDP-induced cell death was reduced by miR-382 inhibition, in contrast overexpression of miR-382 increased cell death induced by CDDP in LS1034 cells (Figure 1B). Moreover, LS1034 had higher CDDP-induced cell apoptosis rate by overexpression of miR-382, whereas inhibition of miR-382 significantly reduced the number of apoptotic cells induced by CDDP, this result was confirmed by flow cytometric analysis (Figure 1C). Additional evidence was the same, CDDP-induced cleavage of caspase 3 was decreased by miR-382 knockdown, and miR-382 expression affecting CDDP-induced cleavage of caspase 3 was opposite (Figure 1D).

### MiR-382 suppresses CRC cell growth *in vitro* and *in vivo*

**Figure 2:**
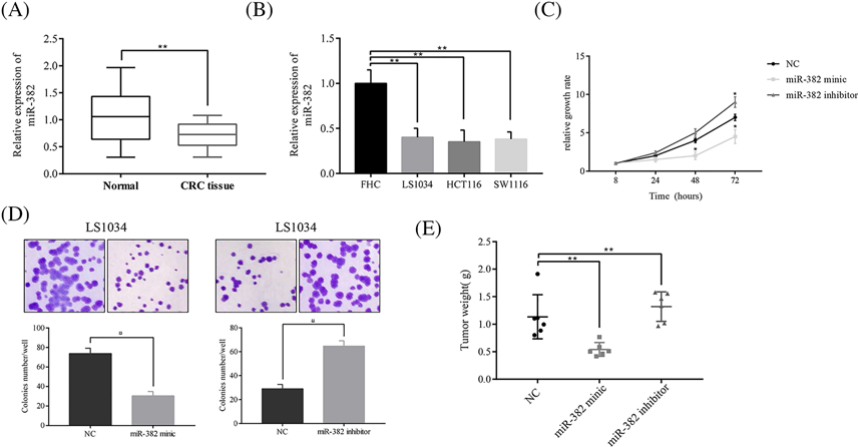
MiR-382 suppresses CRC cell growth *in vitro* and *in vivo* (**A**) MiR-382 expression was decreased in CRC tissue (*n*=55) compared with normal mucosa (*n*=68). (**B**) CRC cell lines show a low expression level of miR-382 compared with normal colonic epithelial cell line FHC. (**C**) Overexpression of miR-382 inhibits LS1034 proliferation, whereas inhibition of miR-382 stimulated LS1034 proliferation compared with the control. (**D**) Inhibition of miR-382 stimulated colony formation, whereas overexpression of miR-382 inhibited colony formation, compared with the control in LS1034 cells. (**E**) Overexpression of miR-382 inhibited tumor growth, whereas inhibition of miR-382 stimulated tumor growth in a LS1034 Xenograft model. The data are presented as the mean ± SD from three independent experiments; *P<0.05 and **P<0.01.

In the cohort of 86 cases of paired CRC tissues and adjacent normal mucosa, miR-382 was significantly down-regulated in tumors compared with the adjacent normal mucosa (Figure 2A). The miR-382 expression level in the CRC cell lines compared with the expression in the normal colonic epithelial cell line FHC. As expected, miR-382 was consistently down-regulated in CRC cell lines compared with FHC cells (Figure 2B).

To better understand the role of miR-382 in CRC, we measured the growth-promoting effect of miR-382 on CRC cells by CCK-8 and colony formation assays after overexpression or inhibition of miR-382 in CRC cells. Overexpression of miR-382 significantly inhibited HCT116 cell growth (Figure 2C). In colony formation assay in another CRC cell line LS1034, we observed that overexpression of miR-382 inhibited LS1034 colony formation, in contrast, inhibition of miR-382 stimulated LS1034 colony formation compared with the control (Figure 2D). These results parallel to those obtained from CCK-8 assays performed on HCT116 cells. In order to determine whether miR-382 was involved in this process *in vivo*, we have used a LS1034 Xenograft model. Overexpression or knockdown of miR-382 inhibited or stimulated tumor growth, respectively, compared with the control group (Figure 2E).

### KLF12 and HIPK3 are targets of miR-382

**Figure 3:**
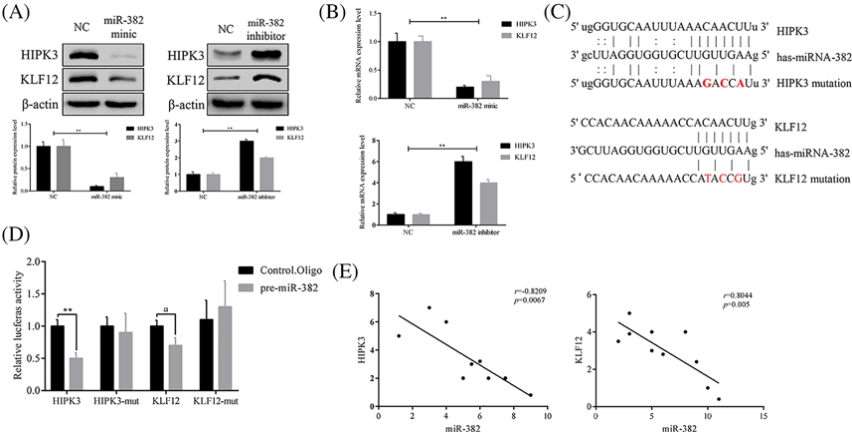
KLF12 and HIPK3 are targets of miR-382 Expression of KLF12 and HIPK3 was negatively regulated by miR-382 at both protein levels (**A**) and *mRNA* (**B**). (**C**) Sequence alignment of miR-382 with the 3′-UTR of the KLF12 and HIPK3 genes. (**D**) KLF12 and HIPK3; HEK293T cells were cotransfected with an indicated pMIR-3′-UTR luciferase reporter construct and pre-miR-382 or respective controls (Ctrl. Oligo). Analysis by 3′-UTR luciferase reporter assay. (**E**) The inverse correlation between miR-382 and KLF12 or HIPK3 mRNA expression in CRC samples (*n*=10) by linear regression analysis; *P≤0.05 and **P<0.01*.*

MiRNAs have been reported to function by binding a specific target, as negative regulators of gene expression regulation. To examine whether miR-382 has a similar mechanism, we overexpressed or inhibited miR-382 and found that KLF12 and HIPK3 protein level, mRNA level were changed with it (Figure 3A for protein level and Figure 3B for mRNA level). As shown in figures, inhibition of miR-382 increased the expression of KLF12 and HIPK3 at both the mRNA and protein level. In contrast, KLF12 and HIPK3 expression were significantly decreased by ectopic overexpression of miR-382 at both the mRNA and protein level. To determine whether the KLF12 and HIPK3 luciferase expression regulation depended on the binding between their complementary 3′-UTR sequences and the miR-382 seed, a three point mutation was inserted in the KLF12 or HIPK3 3′-UTR (Figure 3C), then we transfected the reporter plasmid into HEK293T simultaneously with miRNA-382 mimics or control mimics. We found that miR-382 significantly inhibited the expression of the wild-type luciferase reporter plasmid but had no effects on the mutant plasmid in both cell lines (Figure 3D) together, our data determined that miR-382 negatively regulates the expression of KLF12 and HIPK3 by directly targeting their 3′-UTR sequences. In another way, we demonstrated that the expression of miR-382 and KLF12 or HIPK3 has an inverse correlation in CRC patients (Figure 3E).

### KLF12 and HIPK3 regulated chemoresistance and cell growth in CRC cells

**Figure 4:**
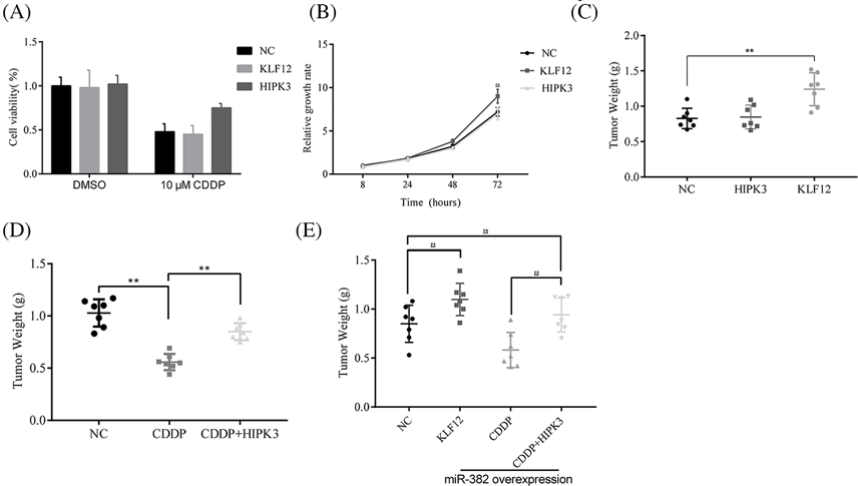
KLF12 and HIPK3 regulated chemoresistance and cell growth in CRC cells (**A**) Overexpression of HIPK3 protected cells from death induced by CDDP treatment. (**B**) Overexpression of KLF12, but not HIKP3, stimulated cell growth. (**C**) In the CRC Xenograft model, tumor growth was increased by KLF12 overexpression, but not by HIPK3 overexpression. (**D**) HIPK3 overexpression induced resistance to CDDP in the LS1034 CRC Xenograft model. (**E**) Overexpression of KLF12 stimulated tumor growth and HIPK3 overexpression induced resistance to CDDP in a miR-382-overexpressing LS1034 Xenograft model; *P≤0.05 and **P<0.01.

**Figure 5:**
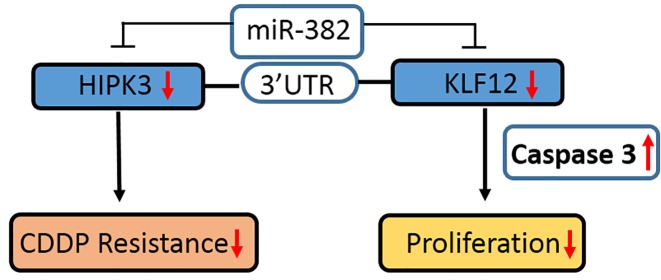
A schematic model of miR-382 as the tumor suppressor and chemosensitizer in colorectal cancer

To determine whether the antitumor effects of miR-382 on these CRC cells could be partly explained by its targeting of KLF12 and HIPK3, we confirmed how KLF12 and HIPK3 overexpression affected cell growth and chemosensitivity *in vitro* and *in vivo*. The data mean that CDDP-induced cell death was reduced by HIPK3 overexpression in HCC2998 cells, but KLF12 did not (Figure 4A); however, KLF12 overexpression significantly stimulated HCC2998 cell growth, and HIPK3 did not (Figure 4B). Beside, the Xenograft model result parallel to the *in vitro* results, KLF12 overexpression stimulated tumor growth and HIPK3 overexpression induced chemoresistance in LS1034 (Figure 4C,D). Then, overexpression of KLF12 and HIPK3 significantly stimulated tumor growth and chemoresistance in miR-382-overexpressing LS1034 cell Xenograft models (Figure 4E). In short, these experiments demonstrated that miR-382 is the major functional upstream regulator of KLF12 and HIPK3, and it controls chemoresistance and cell growth in CRC cells (Figure 5).

## Discussion

CRC was one of the most malignant cancer types in the world, even great advances have been made in the field of CRC treatment in the past decades, CRC death rate was remained high, due to the chemoresistance of CRC to chemotherapeutic drug [11]. Currently, the molecular mechanisms underlying the development and CDDP resistance of CRC are still poorly understood [12]. Thus, it is necessary to determine the molecular mechanisms responsible for the chemoresistance of CRC. It will be valuable to develop novel therapeutic targets and strategies for the treatment.

Previous studies showed that miR-382 involves progression in several types of human malignancy. Decreased miR-382 levels were reverse-correlated with poor outcomes in esophageal squamous cell carcinoma patients [13]. MiR-382 was reported as an angiogenic oncogene in gastric cancer [14]. An inverse correlation existed between aggressive tumor behavior and the residual expression of miR-382 in osteosarcoma [15]. In the present study, we observed a decreased expression of miR-382 in primary CRC tissues compared with normal colon mucosa. Moreover, we found that miR-382 was down-regulated in CRC cell lines compared with a normal colonic epithelial cell line. The data shown that miR-382 could reduce cell growth and enhance chemosensitivity in CRC cells, and *in vivo* experimental results were equal. Collectively with the other report, our results highlight the functional characteristics of miR-382 as a tumor suppressor and chemosensitizer in CRC.

The KLF family represents transcription factors that play important roles in mammalian cells, up to now, 17 subtypes have been identified [16,17]. It affects cell differentiation, proliferation, and apoptosis [18]. Some of them have been reported to act as tumor-suppressors or oncogenes in CRC [16,19,20]. For example, FLF12 promoted gastric cancer cell proliferation and invasion *in vitro* [21], and recent research showed that KLF12 enhancement was found in approximately 40% of esophageal adenocarcinoma cancers [22] and in 45% of salivary tumors [23] and our results demonstrate that KLF12 acts as a tumor-suppressor and chemical sensitizer in CRC and this involved with miR-382, miR-382 overexpression induce KLF12 significantly decreased.

HIPK3 is involved in cell survival and insulin metabolism, and it encodes by the protein homeodomain interacting protein kinase 3 [24,25]. HIPK3 expression level refers to prognosis and sensitivity to chemotherapy, higher HIPK3 expression with badly prognosis and chemoresistance in osteosarcoma and prostate cancer [8,26]. Here, our data show that overexpression of miR-382-induced HIPK3 decreased, which impairs tumor cell survival and induces CDDP-resistant cells acquisition chemically sensitive again and vice versa. These findings further indicate HIPK3 as a possible target to impair tumor metastasis as well as inducing higher sensitivity to chemotherapy in CRC.

In summary, the present study identifies the regulatory link between miR-382 and KLF2/HIPK3 and describes a potential mechanism underlying miR-382 contribution to cell growth and CDDP resistance. The present study demonstrates that the expression of miR-382 is significantly down-regulated in clinical CRC tissues and cell lines, it is negatively associated with KLF2/HIPK3 protein levels. Overexpression of miR-382 suppresses CRC cell growth, CDDP resistance by directly targeting KLF2/HIPK3. Therefore, miR-382 may be considered a tumor suppressor and chemosensitizer that has significant value as an indicator for unfavorable progression in CRC patients and may serve as a therapeutic target in the future.

## Abbreviations

CRC: colorectal cancer
GAPDH: glyceraldehyde-3- phosphate dehydrogenase
HIPK3: homeodomain-interacting protein kinase 3
KLF12: Krüppel-like factor 12
